# Artificial Legs

**Published:** 1874-12

**Authors:** 


*Artificial Legs.*

Should you wish to buy an artificial leg, untainted by partisan views and free from the infirmities of human flesh, see advertisement and write to Dr. Richard Clement, No. 929 Chestnut street, Philadelphia.

